# Case report: Aseptic splenic abscesses in childhood-onset systemic lupus erythematosus

**DOI:** 10.3389/fped.2023.1214551

**Published:** 2023-07-13

**Authors:** Shobashenee Sritharan, Peter Sie-Teck Lau, Kamilah Manan, Anand Mohan

**Affiliations:** ^1^ Department of Pediatrics, Bintulu Hospital, Ministry of Health Malaysia, Bintulu, Malaysia; ^2^ Department of Radiology, Bintulu Hospital, Ministry of Health Malaysia, Bintulu, Malaysia; ^3^ Institute of Health and Community Medicine, Universiti Malaysia Sarawak, Kota Samarahan, Malaysia

**Keywords:** splenic abscess, systemic lupus erythematosus, children, aseptic abscesses, differential diagnosis

## Abstract

**Background:**

Systemic lupus erythematosus (SLE) can involve any organ system and cause a wide range of manifestations. Noninfectious inflammatory lesions termed aseptic abscesses have been reported in some autoimmune and autoinflammatory conditions but not in childhood-onset SLE. In this report, we highlight the unusual finding of occult splenic abscesses in two children diagnosed with SLE who had no evidence of concomitant infection.

**Case presentation:**

An 8-year-old and an 11-year-old were admitted separately to the hospital with fever for 7 and 14 days, respectively. In the younger child, a generalized rash preceded the fever. Both had been well, with no significant past medical history prior to the onset of the illness. In both girls, abdominal ultrasonography showed multiple small hypoechoic lesions suggestive of abscesses scattered throughout the spleen. Their C-reactive protein and blood cultures were negative, and symptoms persisted despite intravenous antibiotics. Fulfilling the clinical and immunologic criteria for diagnosis, both were ultimately diagnosed with childhood-onset SLE. Rapid recovery of symptoms and complete resolution of the abscesses ensued with corticosteroids and immunosuppressive therapy.

**Conclusions:**

These two cases suggest that aseptic splenic abscesses may occur in childhood-onset SLE. Autoimmune conditions such as SLE should be included in the differential diagnosis of children with occult splenic abscesses.

## Background

Systemic lupus erythematosus (SLE) is a chronic autoimmune disease characterized by autoantibody production directed at several self-molecules found in the nucleus, cytoplasm, and cell surface, in addition to soluble molecules (1). The disease can involve any organ system, cause a wide range of manifestations, and lead to significant morbidity and even mortality (2). In addition to the organ damage caused by host-directed antibodies, individuals with SLE are at increased risk of infection due to immunosuppression resulting from both the disease and its treatment (3).

Aseptic abscesses are an emergent and likely underrecognized noninfectious inflammatory disorder characterized by deep, sterile lesions consisting of neutrophils (4). Although the spleen is the dominant organ involved, aseptic abscesses can also affect the abdominal lymph nodes, liver, lung, pancreas, and brain (4). Aseptic splenic abscesses are most frequently reported in autoimmune and autoinflammatory conditions such as Crohn's disease, neutrophilic dermatosis, relapsing polychondritis, and Behcet's disease (4, 5). These abscesses, however, are not a recognized manifestation of SLE. Indeed, splenic abscesses in individuals with SLE are much more likely to be due to infections caused by opportunistic pathogens (6, 7). In this report, we highlight the unusual finding of occult splenic abscesses in two children who were diagnosed with SLE but who had no evidence of concomitant infection, suggesting that the abscesses were of aseptic etiology.

## Case presentation

### Case 1

**Figure 1:**
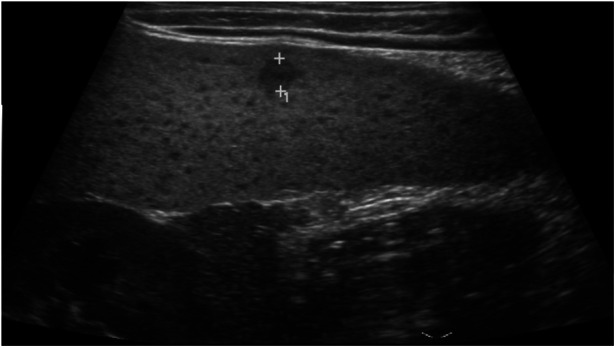
Image of an 8-year-old girl with a skin rash, prolonged fever, and multiple small splenic abscesses who was diagnosed with childhood-onset SLE. Abdominal sonogram showing a 0.5 cm hypoechoic splenic lesion.

An 8-year-old girl presented with an 8-day history of rash and 7 days of fever (Supplementary Figure S1). The rash had started on her face and then spread to her trunk and limbs. The fever was high grade. Her past medical history was unremarkable, and she had no history of environmental exposure to soil or use of untreated water. On Day 6 of her illness, treatment for her symptoms was sought, and she was admitted to a private medical center. Physical examination revealed a generalized erythematous macular rash predominantly affecting the face, multiple enlarged cervical lymph nodes measuring up to 2 cm, and hepatosplenomegaly. Abdominal ultrasonography showed a diffusely enlarged liver with a span of 13 cm and an enlarged spleen with a bipolar length of 11 cm. Additionally, a few ill-defined hypoechoic lesions were found in the spleen, with the largest measuring 0.5 cm (Figure 1). She was started on intravenous ceftriaxone but showed no improvement after 2 days and was subsequently referred to our hospital.

Further examination following admission revealed the presence of a malar rash, livedo reticularis, and oral ulcers. Her antibiotics were switched to intravenous ceftazidime and oral azithromycin. However, the rash and fever persisted after 6 days of antibiotics, and her illness was further complicated with the development of pain and swelling of the right knee.

### Case 2

**Figure 2:**
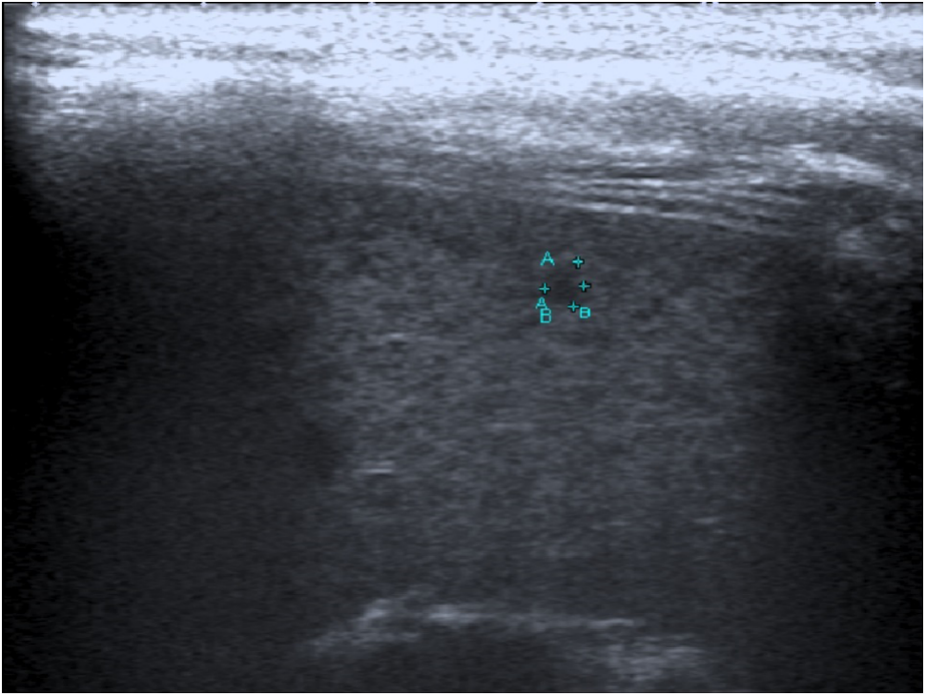
Image of an 11-year-old girl with prolonged fever and multiple small splenic abscesses who was diagnosed with childhood-onset SLE. Abdominal sonogram showing a 0.3 cm hypoechoic splenic lesion.

An 11-year-old girl presented to our hospital with a 2-week history of fever (Supplementary Figure S2). She had received courses of oral amoxicillin and cefuroxime from general practitioners, but without any improvement. The fever was high grade with chills but no rigor. Except for a mild cough and reduced appetite, no other symptoms were present. Her past medical history was unremarkable; she had no history of environmental exposure to soil or the use of untreated water. Physical examination revealed a high-grade temperature and hepatosplenomegaly but no rash, oral ulcers, or alopecia. Abdominal ultrasonography showed a mildly enlarged liver with a span of 14 cm. Several ill-defined hypoechoic lesions were found scattered throughout the spleen, with the largest measuring 0.5 cm (Figure 2). Additionally, a few well-defined rounded soft tissue lesions measuring 1.4 cm were seen at the splenic hilum, likely representing enlarged lymph nodes.

She was initially started on oral erythromycin and intravenous ceftriaxone, but this was switched to intravenous ceftazidime in view of the ultrasonography findings. However, her symptoms persisted after 11 days of antibiotics.

### Investigations, treatment, and outcome in both cases

The investigation results of both cases are shown in Table 1. Both patients fulfilled the criteria for diagnosis of SLE based on the American College of Rheumatology (ACR) and/or the Systemic Lupus International Collaborating Clinics (SLICC), and treatment once laboratory immunological results were available was initiated. Patient 1 fulfilled both the ACR and SLICC diagnostic classification with the presence of 7 of 11 and 10 of 17 criteria, respectively; these included malar rash, photosensitivity, oral ulcers, renal disorder (persistent proteinuria), hematologic disorder (hemolytic anemia, leukopenia and lymphopenia, thrombocytopenia), immunologic disorder (anti-ds DNA antibody above laboratory reference range, presence of anti-Sm antibodies), abnormal titre of antinuclear antibody, and low complement. Patient 2 fulfilled the SLICC diagnostic classification with the presence of 4 (with at least one clinical criterion and one immunologic criterion) of the 17 criteria, including hemolytic anemia, leucopenia and lymphopenia, antinuclear antibody above laboratory reference range, and anti-ds DNA antibody above laboratory reference range. Patient 1 received intravenous immunoglobulin (2 g/kg in total) and methylprednisolone (30 mg/kg/day for 3 days), followed by oral prednisolone, hydroxychloroquine, and azathioprine. Patient 2 received methylprednisolone (10 mg/kg/day for 3 days) followed by oral prednisolone and hydroxychloroquine.

**Table 1:** Laboratory investigation results for two cases with splenic abscesses.

Investigation	Case 1	Case 2
Hemoglobin, g/dl	8.8	9.9
Total white cell count, × 10^9^ cells/L	2.0	1.7
Neutrophil count, × 10^9^ cells/L	1.3	0.9
Lymphocyte count, × 10^9^ cells/L	0.5	0.7
Platelet count, × 10^9^ cells/L	66	373
Erythrocyte sedimentary rate, mm/hr	82	82
C-reactive protein, mg/L	< 10	< 10
Serum ferritin, ng/mL	2,897	937
Total bilirubin, μmol/L	5	7
Direct bilirubin, μmol/L	3	3
Aspartate amino-transferase, U/L	173	38
Alanine amino-transferase, U/L	62	21
Albumin, g/L	36	38
Direct Coombs test	Positive	Positive
Blood culture	Repeatedly negative	Repeatedly negative
Urine protein	1 g/m^2^/day	negative
*Burkholderia pseudomallei* ELISA IgM	Repeatedly negative	Repeatedly negative
Anti-nuclear antibodies	1:1,280; homogenous	1:2,560; homogenous
Anti-double stranded DNA antibodies, IU/ml	> 1,000	430.1
Anti-extractable nuclear antigen antibodies	Positive for Anti-Sm and Anti-U1-RNP	Negative
C3, g/L	0.17 (low)	1.70 (normal)
C4, g/L	0.02 (low)	0.31 (normal)
Bone marrow aspirate	No access of blasts or increased histiocytic activity; culture negative	Hypocellular marrow with reduced granulopoiesis; culture negative

In both children, resolution of fever occurred within a day of methylprednisolone initiation, with rapid resolution of all other symptoms within the following 3 weeks and gradual normalization of hematological parameters. Patient 1 completed a total of 2 weeks of ceftazidime and was subsequently started on oral cotrimoxazole, which was discontinued once the result of her paired *Burkholderia pseudomallei* serology was reviewed. For Patient 2, all antibiotics were discontinued upon initiation of methylprednisolone. In both cases, repeat abdominal ultrasonography performed after 2 months showed complete resolution of the splenic lesions. Both children are currently asymptomatic, with good control of disease activity on oral corticosteroids and immunosuppressive therapy.

## Discussion and conclusions

To the best of our knowledge, this is the first report to describe splenic abscesses in childhood-onset SLE. A search of the PubMed/MEDLINE database using the key terms “spleen abscess”, “splenic abscess”, “systemic lupus erythematosus”, and “SLE” revealed no reports of similar cases in the published literature. Although only a limited infectious disease work-up was performed in our two cases, no evidence of a concomitant infectious process or disease was found. All clinical and laboratory findings were, however, consistent with the diagnosis of SLE, and rapid recovery and complete resolution of the abscesses ensued with corticosteroids and immunosuppressive therapy.

The diagnosis of SLE was confirmed in both cases by the presence of clinical and immunologic criteria fulfilling the ACR and/or SLICC classification criteria for SLE (8–10). Although SLE occurs less frequently in children than in adults, the true incidence and prevalence of childhood-onset SLE in many regions remain unknown due to the absence of nationwide population-based epidemiological studies (11). Childhood-onset SLE is, nevertheless, reported to be more severe than SLE in adults (12). Clinical characteristics, disease activity, and outcome may, however, differ between different ethnic groups (13). As in adults, diagnosis is based on the presence of suggestive clinical features supported by laboratory findings. Various diagnostic criteria have been developed primarily for research purposes but are often used to aid clinical diagnosis. For example, the use of the ACR criteria is associated with a sensitivity and specificity of >95% for diagnosis of childhood-onset SLE (14). Although not part of the diagnostic criteria, involvement of the reticuloendothelial system organs is common (15). In adults, reported splenic manifestations include diffuse enlargement, rupture, infarcts, granulomas, calcifications, atrophy, and hypofunction (15–20).

For both children reported herein, multiple occult splenic abscesses were detected by abdominal ultrasonography during the initial work-up after presenting with prolonged fever. No evidence of infection was found, and symptoms persisted despite empirical antibiotics. Splenic abscesses represent a rare disease entity in childhood. Small dispersed lesions in the spleen, sometimes described as Swiss cheese in appearance, are usually caused by either bacterial (melioidosis, mycobacterial, cat-scratch disease, brucellosis, infective endocarditis, Salmonella), protozoan (leishmaniasis), or mycotic (Candida, Aspergillus, Cryptococcus, Pneumocystis) infection or malignancy (lymphoma) (21–26). As our hospital is in a melioidosis-endemic region, abdominal ultrasonography (to detect splenic abscesses) is routinely performed in all children presenting with prolonged fever, as it has been shown to facilitate the diagnosis of melioidosis (24). We routinely use a high-frequency linear transducer in the imaging protocol, enabling detection of very small lesions (measuring <5 mm) not normally detected by conventional curvilinear transducers (22). These local protocols likely inadvertently led to the detection of occult splenic lesions in the two children described in this report. Although both children were initially empirically treated for melioidosis, by far the most common cause of splenic abscesses in our hospital (24), neither had risk factors for *Burkholderia pseudomallei* acquisition, and both were negative for C-reactive protein, with negative blood cultures and melioidosis serology; they did not respond to melioidosis-active antibiotic regimens and were ultimately diagnosed as having SLE.

The ultrasonography findings in our two cases may thus represent aseptic splenic abscesses, an association or manifestation not previously reported in childhood-onset SLE. In adults and adolescents, splenic lesions resembling abscesses or granuloma but without any evidence of infection have rarely been reported in association with SLE. For example, a 15-year old boy was found to have a single hypoechoiec splenic lesion following presentation with prolonged fever and generalized body swelling and was initially thought to have disseminated tuberculosis but was ultimately diagnosed with SLE (16). Additionally, hepatic lesions resembling abscesses that responded to corticosteroids but not to antibiotics have also been reported (27, 28). Nevertheless, as biopsy of the lesions was not performed in our two cases, further studies are needed to confirm our findings and to determine the prevalence of these abscesses in childhood-onset SLE. This is especially important because detection of any abscess in individuals with SLE will typically lead to a thorough (and appropriate) search for infection and probable empirical antibiotic treatment but may cause an inappropriate delay in the initiation of corticosteroids and immunosuppressive therapy and unnecessary use of antibiotics if the abscesses truly represent aseptic lesions.

In conclusion, we report the rare finding of splenic abscesses in two children with SLE. No evidence of concomitant infection was found, and the symptoms and splenic lesions resolved with corticosteroids and immunosuppressive therapy. These cases suggest that aseptic splenic abscesses may occur in childhood-onset SLE. Autoimmune conditions such as SLE should be included in the differential diagnosis of children presenting with occult splenic abscesses.

## Data Availability

The original contributions presented in the study are included in the article/**Supplementary Material**, further inquiries can be directed to the corresponding author.

## Supplementary material

Supplementary Figure S1 Timeline of clinical and diagnostic events for Patient 1.

Supplementary Figure S2 Timeline of clinical and diagnostic events for Patient 2.
