# Supplementary figures and images for: Case report: Aseptic splenic abscesses in childhood-onset systemic lupus erythematosus
Source: Front Pediatr. 2023 Jul 13;11:1214551. doi: 10.3389/fped.2023.1214551 (PMC10374253; doi:10.3389/fped.2023.1214551)

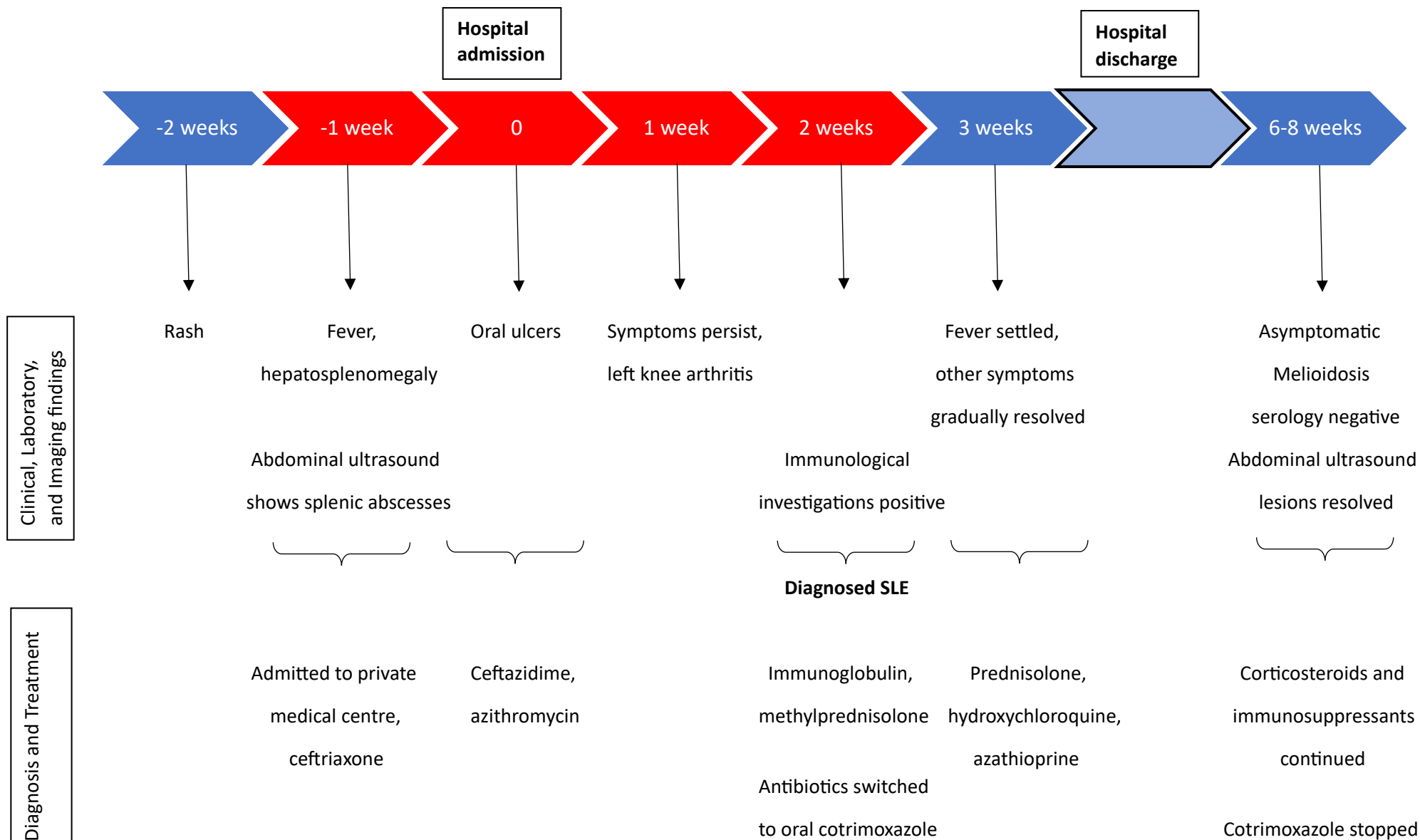

Supplement: Supplementary Figure S1 — Timeline of clinical and diagnostic events for Patient 1. [file Image1.pdf]

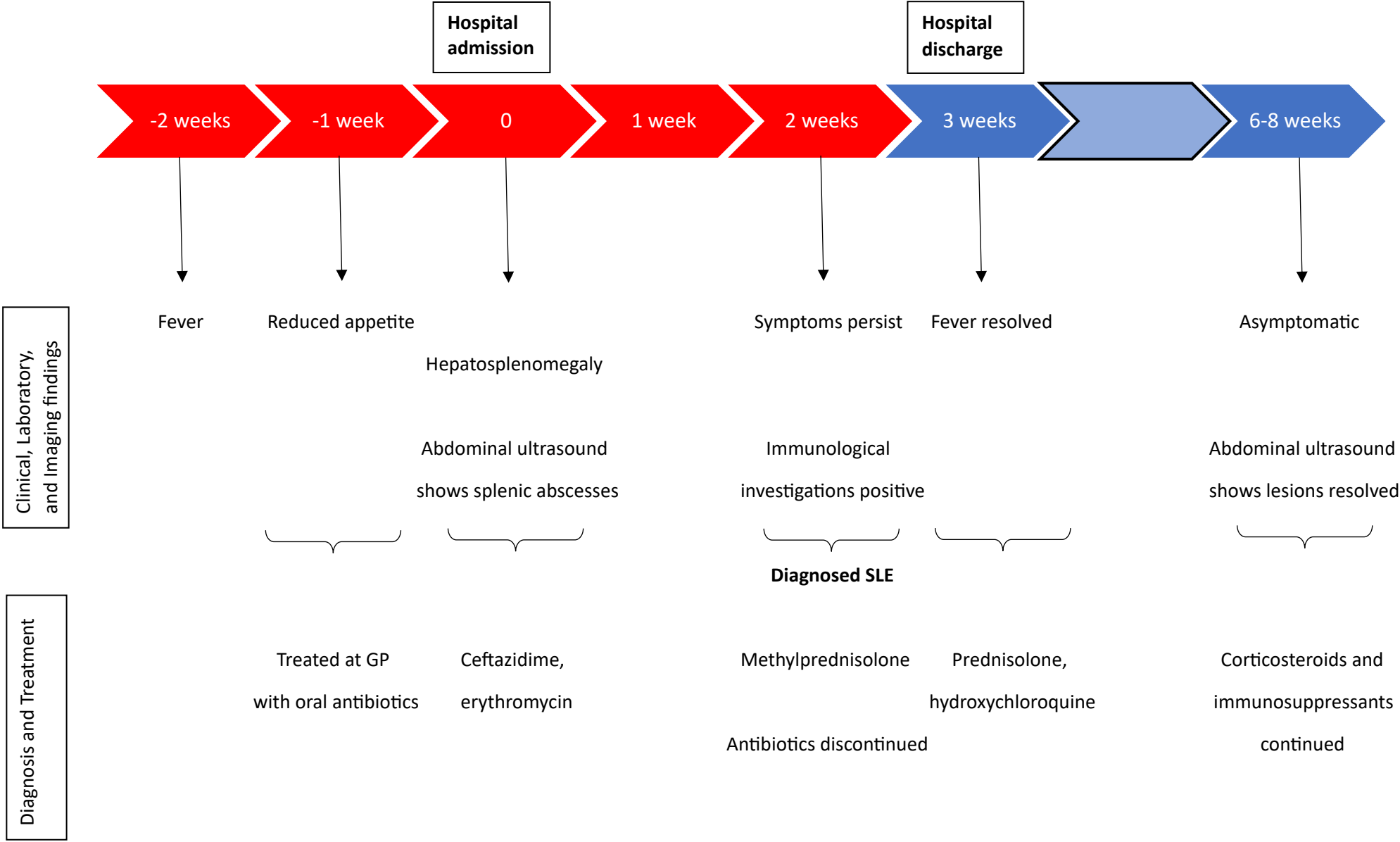

Supplement: Supplementary Figure S2 — Timeline of clinical and diagnostic events for Patient 2. [file Image2.pdf]
